# Importance of demographic surveys and public lands for the conservation of eastern hellbenders *Cryptobranchus alleganiensis alleganiensis* in southeast USA

**DOI:** 10.1371/journal.pone.0179153

**Published:** 2017-06-08

**Authors:** Michael J. Freake, Christopher S. DePerno

**Affiliations:** 1 Department of Natural Sciences and Mathematics, Lee University, Cleveland, Tennessee, United States of America; 2 Department of Forestry and Environmental Resources, Fisheries, Wildlife, and Conservation Biology Program, North Carolina State University, Raleigh, North Carolina, United States of America; Charles University, CZECH REPUBLIC

## Abstract

Comparisons of recent and historic population demographic studies of eastern hellbenders *Cryptobranchus alleganiensis alleganiensis* have identified significant population declines and extirpations associated with habitat degradation, poor water quality and disease, leading to nomination as a candidate for listing under the Endangered Species Act. However, populations in the southern Appalachian region of the range have received less attention despite relatively high levels of watershed protection due to the establishment of federally protected National Forest and National Park public lands. These watersheds likely represent some of the best remaining available habitat, yet the lack of published studies make assessment of population stability and viability very difficult. Our objectives were to (1) conduct a capture-mark-recapture (CMR) demographic study and a point transect survey on the Hiwassee River in Tennessee which is designated a National Scenic River, and is largely contained within the Cherokee National Forest, (2) quantify the size structure of the population, (3) compare abundance, survival and recruitment with historic and contemporary hellbender populations across the range, (4) assess the importance of this population and the significance of National Forest and National Park lands in the context of hellbender population conservation in the southeastern United States. We detected all age classes present, with larval hellbenders comprising 21.5% of captures. Using a combination of static life table and CMR methods, we determined that survival rates during the first year were low (~10%), but were high (68–94%) for taggable sized hellbenders. Density of hellbenders at the study site was very high (84 taggable sized hellbenders per 100m of river) compared to recent demographic studies conducted in other regions of the range. We detected hellbenders over ~28 km of river, with a mean density of 23 taggable sized hellbenders per 100m of river, and a total population estimate of 6440 taggable hellbenders. National Forest and National Park lands are likely to continue to play a particularly important role in providing suitable habitat for hellbenders in the southern Appalachians. In fact, only six of 21 known hellbender locations in Tennessee appear to show consistent larval recruitment, all of which are located within or adjacent to National Forest or National Park land.

## Introduction

Hellbenders (*Cryptobranchus* spp.) are giant aquatic salamanders (family Cryptobranchidae) with an extensive historic distribution in the USA [1]. The eastern hellbender (*C*. *alleganiensis alleganiensis*) ranges from southern New York to northern Georgia, Alabama and Mississipi, with disjunct Midwest populations in Missouri. The Ozark hellbender (*C*. *alleganiensis bishopi*) range is limited to the Ozark region of Arkansas and Missouri. Across their range, hellbenders populations have declined significantly because of numerous factors including river impoundment, poor water quality and siltation, persecution, illegal collection, and disease [2–6]. The limited historic range, and evidence of dramatic declines [7,8], led to listing of the Ozark hellbender subspecies under the Federal Endangered Species Act (ESA) in 2011 [9] and concerns over range-wide declines in eastern hellbenders resulted in their nomination as a candidate for listing under the ESA in 2013. Both species are included in appendix III of the Convention on International Trade (CITES) in Endangered Species of Wild Fauna [10], and have a IUCN “near-threatened” status. In Tennessee, eastern hellbenders are listed by the state as a Species of Greatest Conservation Need [11].

The ecology of hellbenders presents local and range wide research challenges because they are secretive habitat specialists, typically occupying cool, clear streams with large rocks or crevices that provide shelter, feeding and nest sites [12]. Hellbenders take 5–8 years to reach reproductive maturity and may live 25 or more years [5,12,13]. This combination of longevity and iteroparity, secretive behavior, and the physical environment they occupy requires a very high investment in survey effort over many years to reliably estimate demographic parameters such as abundance, stage specific survival, recruitment and fecundity. These parameters are typically estimated through capture-mark-recapture (CMR) surveys, and are essential for constructing reliable stage structured matrix and population viability models [14–16], which can inform wildlife managers about population status, viability, and effective management strategies.

**Fig 1:**
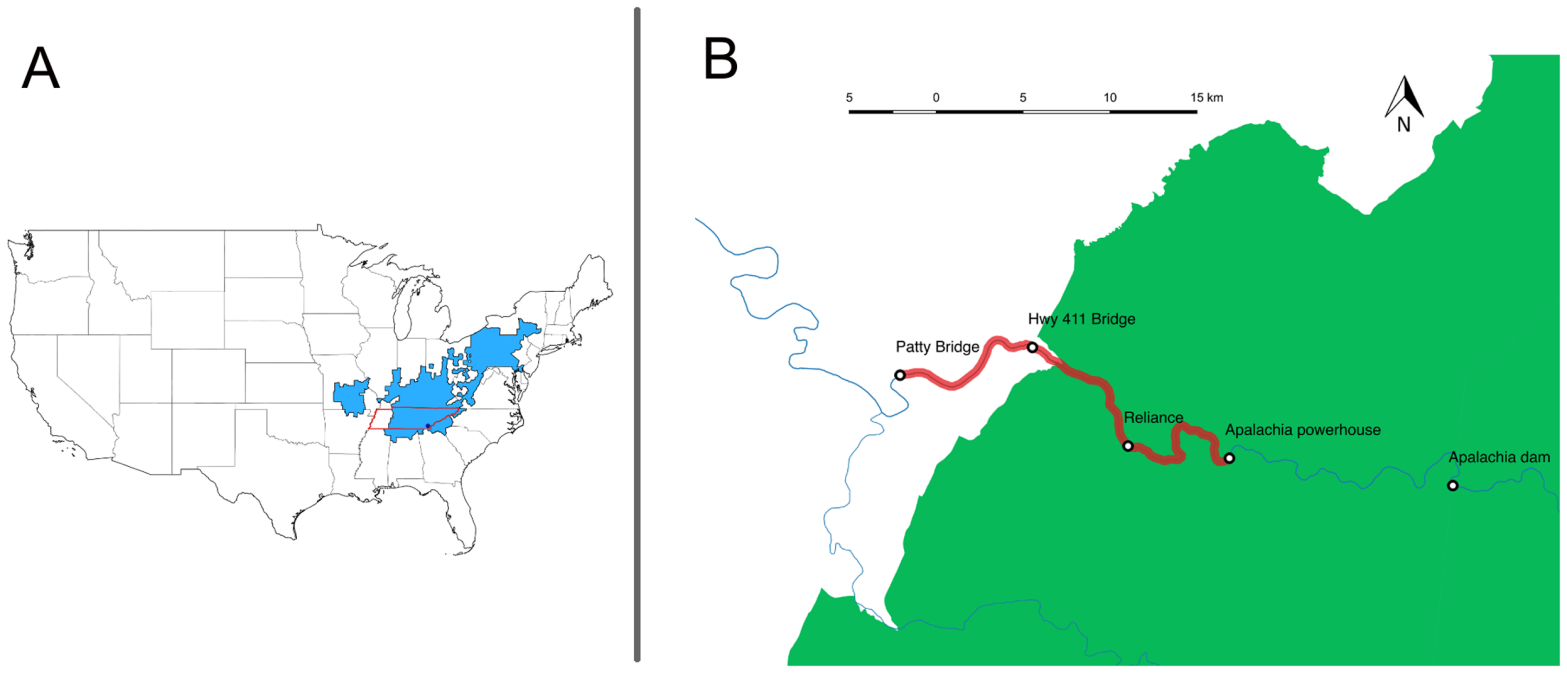
Study area. (A) Range map of hellbenders. Light blue shaded area indicates the distribution of hellbenders *Cryptobranchus alleganiensis*, used with permission from the IUCN Red List [38]. The Tennessee border is indicated in red, and the Hiwassee study site location is shown by a dark blue filled circle. (B) Hiwassee river with the study area highlighted in red. Shaded green area corresponds to National Forest public lands. Detailed location information is withheld to limit the risk of disturbance or illegal collection (see Ethics and Permits below).

The inherent challenge of conducting these types of studies has resulted in a paucity of long-term CMR studies of hellbenders, and a disparity in geographic representation. Most demographic studies have focused on Ozark and eastern hellbender populations in Arkansas and Missouri [5,12,13,17–19], or northern populations of eastern hellbenders [20–23], and can be roughly separated into pre-1990 studies of apparently healthy populations (although see [24]) and post-1990 studies of declining populations. Only two studies compared demographic parameters pre and post decline, revealing a demographic shift towards predominantly large old individuals, a decline in body condition, and overall reduction in abundance of both hellbender subspecies [8,22]. Conversely, a significant gap in demographic studies exists for eastern hellbender populations towards the southern edge of their range in the Tennessee river watershed (Fig 1A), even though large areas of Tennessee, North Carolina and Georgia drain the Tennessee river and historically have provided suitable habitat for eastern hellbenders [25].

Moreover, significant sections of the Tennessee River headwaters and associated watersheds are located within National Forest and National Park public lands, which may provide protection from declining water quality found elsewhere across the range [3,26]. The National Forest Service was established in 1905 with the mission “to sustainably manage the health, diversity, and productivity of the nation’s forests and grasslands to meet the needs of present and future generations”. The National Park Service was established in 1916 with a mission to “preserve unimpaired the natural and cultural resources and values of the National Park System for the enjoyment, education, and inspiration of this and future generations”. As a result, 3700 km^2^ of eastern Tennessee forests are under federal conservation management. An analysis of multiscale factors influencing eastern hellbenders in the northern part of the range [27] indicated that hellbender occupancy was strongly predicted by large substrate size, low embeddedness by sediment, and high percent forest cover, all of which are characteristic of watersheds associated with federal public lands in Tennessee. In contrast, loss of forest cover increases flow rates and sediment inputs [28], and contributes to a decline in stream biodiversity [29]. Nevertheless, even public lands within the southern Appalachian experienced large-scale historic deforestation and mining, and the entire Tennessee Valley was greatly altered by dam construction, especially by the Tennessee Valley Authority from 1933 to 1979 to control floods and generate electricity. These impoundments have caused a loss of suitable habitat, and contribute to isolation and fragmentation of populations in the southeastern United States.

The paucity of hellbender demographic studies in the southeast USA makes assessment of long term abundance and distribution trends difficult, however there is evidence for substantial regional declines. For example, Gentry [30] described eastern hellbenders as widespread and abundant in Tennessee, whereas, Miller and Miller [31] detected only large adults with high rates of physical abnormalities in four Cumberland and Tennessee populations in the Highland Rim physiographic province of middle Tennessee. Recent surveys indicate virtual extirpation of three of these populations (B. Miller and D. McGinnity, Middle Tennessee State University, Nashville Zoo, unpublished data). However, surveys in the Blueridge physiographic province identified healthy populations with successful recruitment within the Cherokee National Forest and Great Smoky Mountains National Park [32–34]. Concerted survey efforts over the last decade in North Carolina [35] and Georgia (L. Williams and J. Humphries, North Carolina Wildlife Resource Commission; J. Groves, Curator Emeritus, NC Zoo; T. Floyd, Georgia Department of Natural Resources; unpublished data) indicate the presence of several populations with relatively high densities of hellbenders and consistent reproduction, suggesting the southern Appalachians may represent one of the most important regions for preserving eastern hellbenders in the United States.

A lack of range-wide detailed historic and contemporary demographic data negatively impacts effective management of declining species, which is exacerbated for secretive species such as hellbenders. Therefore, our objectives were to conduct a five-year CMR survey of an unstudied Tennessee population, quantify the size structure of the population, and compare abundance, survival and recruitment with historic and contemporary hellbender populations across the range. Also, we wanted to assess the importance of this population and the significance of National Forest and National Park lands in the context of hellbender population conservation in the southeastern United States. We expected populations located within or adjacent to protected federal public lands to generally have higher long-term persistence and viability than populations residing in watersheds that have been, and continue to be, modified greatly by anthropogenic forces.

## Methods

### Study area

We conducted our study on the Hiwassee River (elevation 230 m) within a section designated as a Tennessee State Scenic River, located near Reliance Tennessee (Fig 1A). This section is downstream from the Apalachia powerhouse, a bypass hydroelectric system with a flume carrying cold hypolimnetic water 13 km from Apalachia dam to the powerhouse [36, 37]. Between the dam and powerhouse is a warm dewatered section that our surveys have indicated is unsuitable for hellbenders, while below the powerhouse hellbenders have been located for at least 28 km downstream. Most of the river between Hwy 411 and Apalachia powerhouse lies within the Cherokee National Forest, while the entire river from Hwy 411 to Patty Bridge runs through private land largely consisting of crop and livestock agriculture. This lower section tends to exhibit degraded riparian zones characterized by loss of native streamside shrubs and trees coupled with exposed bare soil, causing increased sediment input (Fig 1B).

For further information on the study area, see S1 Appendix.

### Focal site surveys

During 2004 to 2008, we conducted annual surveys of a 350 m section of the Hiwassee near Reliance, Tennessee using standard mask and snorkel rock lifting methods [39]. Typically, there were 3–6 people conducting the surveys. The number of survey days varied among years (5–10 days), and took place between April 30 and September 17, with most surveys conducted in May. We lifted rocks by hand or with a peavey. To increase the probability of catching all size classes, we surveyed extremely methodically, turning all rocks including areas of cobblestone. We calculated catch per unit effort (CPU) as number of hellbenders encountered per person search hour.

For every hellbender we captured, we recorded location (using GPS), mass, total and snout-vent length, and then injected a passive integrated transponder (PIT) tag subcutaneously at the base of the tail in all individuals >15 cm total length (TL). We classified individuals as larvae (<9 cm), subadults (9–29 cm) or adults (≥30 cm). We noted any injuries such as missing toes or legs and collected toe or tail tip tissue samples. In 2005–2006, we recorded shelter rock size (maximum horizontal dimension). We were unable to reliably sex individuals caught before July because Hiwassee hellbenders mate in October (D. McGinnity pers. comm.) and males do not exhibit any cloacal swelling. However, for an additional 82 capture records during July to October 2004–2015, we were able to assign sex based on cloacal swelling.

We used one-way ANOVA followed by Tukey’s honest significant difference test to determine differences between mean shelter rock size used by larvae, subadults, and adults. We conducted simple linear regression to investigate the relationship between shelter rock size and hellbender total length. The analyses were conducted using R version 3.3.1 base statistics package [40].

We used a static life table approach to estimate the survival rate of hellbenders during their first year. From our field observations and previous studies [13,19,32] on growth rates and size classes we considered all gilled hellbenders <9 cm TL to be recently emerged from the nest rock, while all 9–12 cm TL hellbenders were considered to be one year olds. Beyond this age, variation in growth rates make identification of cohorts difficult, and so we relied on capture-mark-recapture methods to estimate survival probabilities of larger hellbenders

### Capture-mark-recapture (CMR)

During the 2004–2008 CMR analysis, we only included captures from the Hiwassee core area that was surveyed from April 30 to June 4 ensuring the survey area was constant from year to year, and sampling occasions were effectively instantaneous [41].

We used the POPAN parameterization of the Jolly-Seber (JS) model [42,43] in program Mark [44] to estimate abundance (*N*_*i*_), and survival (φ_*i*_), capture (*p*_*i*_) and entry probabilities (*b*_*i*_) at time *i*. The POPAN version hypothesizes a “superpopulation” consisting of the total number of unique animals available for capture, with parameters *b*_*i*_ which represent the probabilities that an animal from this hypothetical superpopulation enters the population between occasion *i* and *i* +1 and survive to the next sampling period [45]. We believe that our data meets the assumptions for the Jolly-Seber model including: (1) capture probabilities are the same for all individuals (marked and unmarked) at each sampling occasion, and (2) survival probabilities are the same for all individuals (marked and unmarked) between each pair of sampling occasions.

We performed goodness of fit tests of the JS POPAN model using program RELEASE which produced χ^2^ values for two tests: TEST 2 evaluates assumption 1 by determining if every marked animal present in the population is equally catchable at time (*i*+1) regardless of whether they were captured at time (*i*). Assumption 1 is sensitive to capture effects, or non-random temporary emigration. TEST 3 evaluates assumption 2 by determining if the probability that an individual is ever seen again depends on whether it was marked at or before occasion (*i*). Assumption 2 is sensitive to changes in mortality over the course of the study, or to differences in survival probabilities among individuals (for example individuals of different ages). Also, RELEASE provides a TEST 2 + TEST 3 overall summary χ^2^ test, which can be used to generate an estimate of dispersion (ĉ), given by ĉ = χ^2^/*df*, where ĉ should approximate to 1 if the data fit the model adequately.

We fit JS POPAN models using the logit link function for *φ* and *p*, the log function for *N*, and the multinomial logit link function for *b*. A full parameterization of the JS model includes time dependence for survival and recapture probabilities, so we tested reduced parameter versions with constant survival and/or recapture probabilities. We ranked the models by Akaike’s information criterion AIC_c_ for low sample sizes [46], and then used model averaging which considers all of the models but weights the estimates based on AIC_c_ weight to produce survival, recapture and entry probabilities, and estimates of abundance.

It is possible that different survival probabilities or tendencies to move between subadults and adults could violate the assumptions and reduce the reliability of parameter estimates. Therefore, we performed the same POPAN JS and goodness of fit tests using two groups (subadults 15-30cm, adults >30cm). We failed to detect substantial differences in survival and capture probabilities between subadults and adults, and although goodness of fit tests indicated no deviations from model assumptions (TEST 3: χ^2^ = 3.2707, df = 8, p = 0.9162; TEST 2: χ^2^ = 2.329, df = 3, p = 0.5068; TEST 2 + TEST 3: χ^2^ = 5.6005, df = 11, p = 0.8986), low sample size warnings were returned for several test components, reducing our confidence in the model estimates. Therefore, we believe that pooling all captures maximized precision and reliability of the survival and recapture parameter estimates.

### Point surveys

During 2009 to 2014, we conducted point surveys along the river between the Apalachia powerhouse and Patty Bridge (a total of 28 river km). Points were mapped 500 m apart (powerhouse to Hwy 411) or 750 m apart (Hwy 411 to Patty Bridge) and picked randomly for sampling. We surveyed for 1 hour or for 100m, whichever occurred sooner and recorded CPU. Unlike the CMR study, we did not attempt to methodically turn every rock in the section because the intention was not to characterize the population size structure at each site, but rather to produce relative estimates of abundance of taggable sized hellbenders based on capture rate (hellbenders per person hour). Therefore, we limited our search to rocks >25 cm width. In August 2009, we surveyed the CMR site using this same survey methodology to generate a capture rate that would then be comparable to the other point survey sites. Because we have an estimate of the actual population density at the CMR site from the 2004–8 study, it is possible to use the mean capture rate from the point surveys to estimate the average population density over the entire river, assuming similar detectability at the point survey sites.

### Public lands and hellbender populations

We investigated the importance of public lands for contemporary eastern hellbender populations by accessing all verified observations of eastern hellbenders across Tennessee from the Tennessee Wildlife Resource Agency State Wildlife (TWRA) Action Plan database maintained by the Tennessee chapter of the Nature Conservancy (http://nature.org/). Using qGIS version 2.14 we mapped contemporary (since 2000) hellbender populations and calculated the number of river kilometers that are currently likely to hold hellbenders. Also, we mapped historic hellbender locations that have not produced any recent (since 2000) occurrence observations, and are likely to be functionally extirpated. We used National Park Service, USDA National Forest Service, and TWRA Wildlife Management Areas boundary layers to qualitatively examine the landscape context of recent and historic hellbender occurrences.

### Ethics and permits

This study was performed with the approval of the Institutional Review Board of Lee University. We received permission from the USDA National Forest Service to conduct fieldwork in the Cherokee National Forest. Animals were collected under permit from the Tennessee Wildlife Resource Agency (# 1505), processed and released unharmed at the capture site in accordance with the Guidelines for Use of Live Amphibian and Reptiles in Field and Laboratory Research (American Society of Ichthyologists and Herpetologists, http://www.asih.org/sites/default/files/documents/resources/guidelinesherpsresearch2004.pdf). To protect hellbenders from illegal collection and disturbance [3], publication of detailed location data is prohibited by the state and federal permitting agencies. However these data are available upon request from Bill Reeves, Tennessee Wildlife Resource Agency for approved research use.

## Results

### Captures and size classes

**Fig 2:**
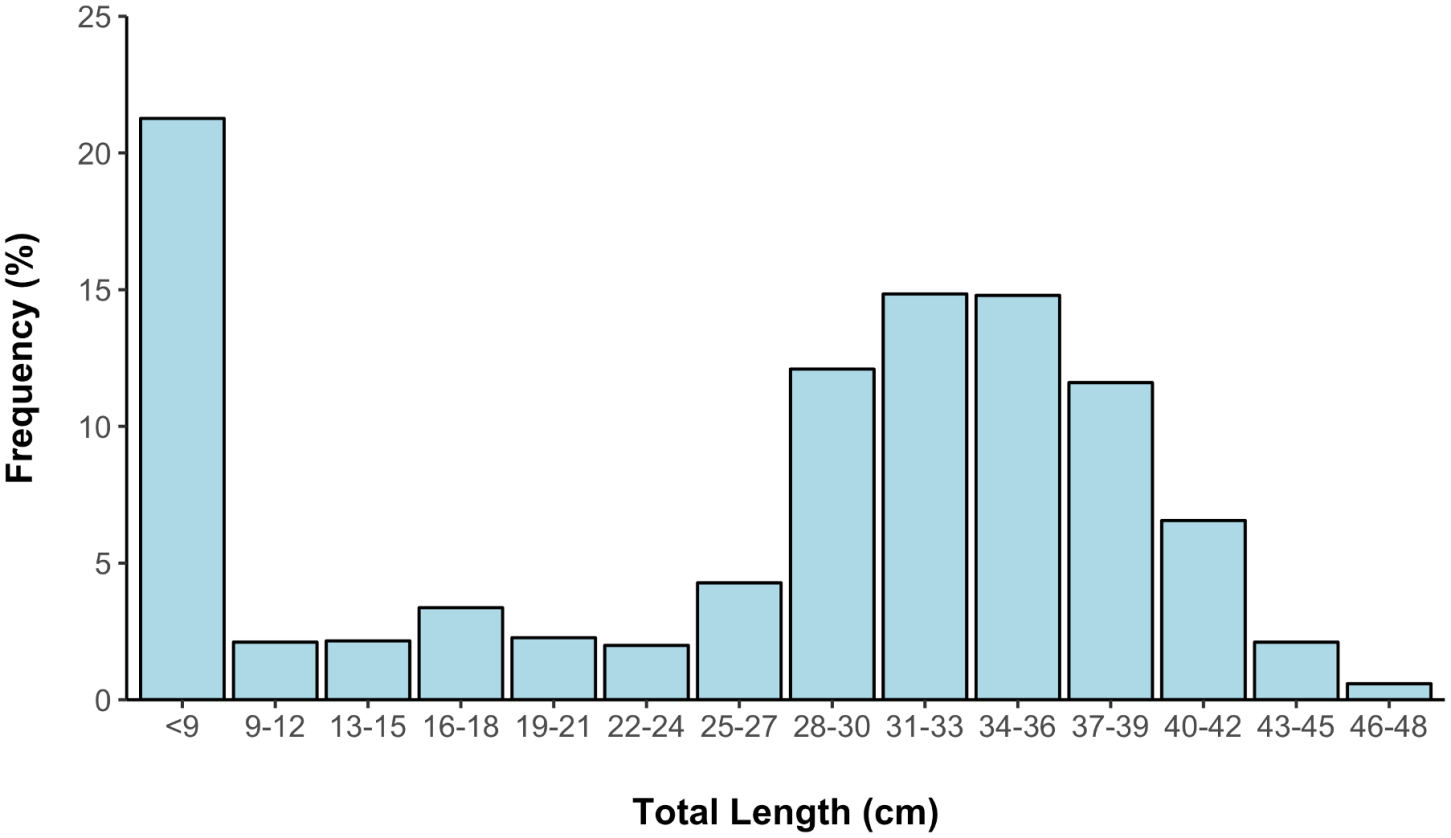
Size frequency distribution of eastern hellbenders (n = 466) averaged over annual surveys conducted 2004–2008, Hiwassee River, Tennessee.

During the 2004–2008 focal site surveys, we captured 466 hellbenders with a CPU = 0.97 hellbenders per person hour. Hellbender sizes ranged from 5 cm gilled larvae to 46 cm adults. All size classes were present (Fig 2): gilled larvae composed 21.5% of the captures while sub-adults and adults composed 28% and 50% of the captures respectively. Of the 82 hellbenders captured July-October 2004–2014 that could be sexed, 35 were males and 47 were females (ratio = 1:1.34). Survivorship of first year hellbenders varied from 0.07 to 0.15 (mean = 0.098).

### Rock use

**Fig 3:**
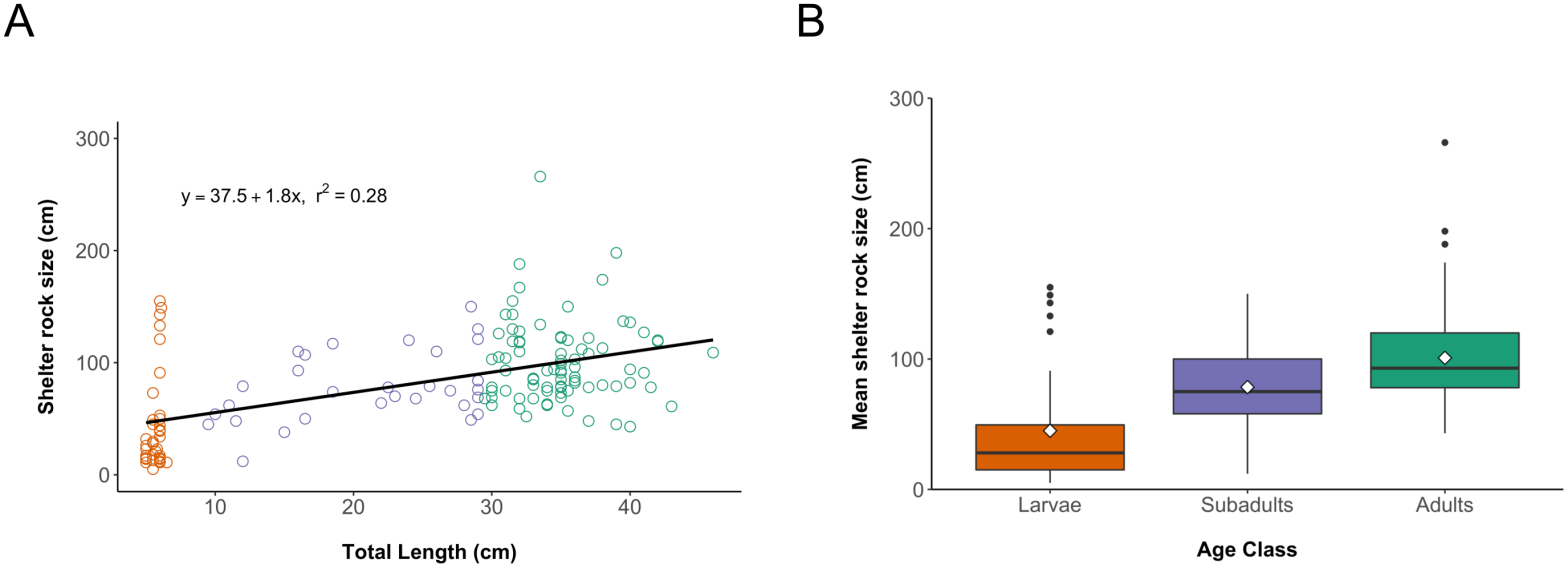
Shelter rock use by eastern hellbenders in the Hiwassee River, Tennessee, 2004–2008. (A) Regression analysis indicates a significant relationship (p < 0.001) between hellbender total length and maximum horizontal dimension of shelter rock (n = 147). (B) Box plots illustrating shelter rock size used by larvae (n = 35), subadults (n = 31) and adults (n = 81). Box boundaries indicate interquartile ranges, heavy line within box indicates the median, whiskers indicate 1.5x interquartile range and solid black dots indicate outliers. The mean is illustrated with white diamonds, and arrows between boxes illustrate pairwise comparisons using Tukey’s honest significant difference test.

We detected a significant linear relationship between body size and rock size (Fig 3A). Rocks used by larvae (mean = 45 cm, 95% CI = 30.0–60.0 cm) tended to be significantly smaller than rocks used by subadults (mean = 78.5 cm, 95% CI = 67.3–89.7 cm) which tended to use significantly smaller rocks than adults (mean = 100.9 cm, 95% CI = 92.7–109.1 cm: ANOVA, F = 27.31, df = 2, p<0.0001, Tukey test: larvae vs subadults, p<0.01; larvae vs adults, p<0.01; subadults vs adults, p<0.05, see Fig 3B). Larvae were most often detected in shallow moderate flow glides consisting primarily of a cobblestone bottom.

### Capture-mark-recapture population estimates

A total of 197 individual hellbenders provided capture records that met the criteria for consistent survey area and time (Table 1). We detected no evidence of significant deviation from the model assumptions (TEST 2: χ^2^ = 3.4621, df = 2, p = 0.1771; TEST 3: χ^2^ = 3.5934, df = 5, p = 0.6093; TEST 2 + TEST 3: χ^2^ = 7.06, df = 7, p = 0.42). The estimate of dispersion ĉ = 1.01, indicating good model fit.

**Table 1:** Capture-recapture matrix for Hiwassee hellbenders caught and released within the focal site core area, Hiwassee River, Tennessee, 2004–2008.

Survey year	Number marked	Recapture year				
		2005	2006	2007	2008	Total
2004	53	11	5	7	4	27
2005	43		7	6	0	13
2006	48			6	7	13
2007	78				9	9

The best supported model was φ(t),*p*(.),*b*(t), where survival varied with time, and recapture probability was constant (Table 2); however the ratio of AIC_c_ weights between the top two models was only 1.46, suggesting little difference in support for the two models. POPAN model averaging (Table 3) provided estimates of survival ranging from 0.68–0.94 (mean = 0.85), capture rates ranging from 0.17 to 0.51 (mean = 0.26), and entry ranging from 0.23 to 0.25 (mean = 0.24). The estimated superpopulation abundance was 405 hellbenders (95% CI = 317–493). The estimated net population varied from 166 to 347 (mean = 243, 95% CI = 156–330). Actual survey area was 7605 m^2^, but to account for hellbender home ranges that only partially overlap the study area, we added buffer strips [47] given by half mean maximum distance moved between recaptures where appropriate. For example, no strip was added to edges of the survey area that extended to the riverbank. The buffer expanded the survey area to 10498 m^2^, producing a density estimate of 2.31 (95% CI = 1.49–3.14) hellbenders per 100 m^2^ of suitable habitat. There were approximately 12745 m^2^ of suitable habitat within the 350 m study section, providing an estimated total mean abundance of 294 hellbenders (95% CI = 190–400), or 84 (95% CI = 54–114) hellbenders per 100 m of river length. These estimates are conservative because hellbenders that are too small to tag comprised 21.5% of all captures at the study site.

**Table 2:** POPAN parameterization of Jolly-Seber model rankings, Hiwassee River, Tennessee, 2004–2008. Parameters are survival (φ), capture (p) and entry (b) probabilities. The parameters may be time dependent (t) or constant (.). Only the four best supported models are shown.

Model	AIC_c_	ΔAIC_c_	AIC_c_ weights	Model likelihood	Number of parameters	Deviance
φ(t),*p*(.),*b*(t)	405.547	0	0.468	1	10	-544.715
φ(.),*p*(t),*b*(t)	406.310	0.763	0.319	0.682	11	-546.133
φ(t),*p*(t),*b*(t)	407.780	2.231	0.153	0.328	12	-546.865
φ(.),*p*(.),*b*(t)	409.649	4.102	0.060	0.129	7	-534.172

**Table 3:** Population parameter estimates and 95% confidence intervals derived from POPAN Jolly-Seber model-averaged estimates. Parameter estimates include apparent survival, capture probability, superpopulation size, net abundance, and entry probability. Hiwassee River, Tennessee, 2004–2008.

Parameter	Year	Estimate	SE	95% Confidence Intervals
Survival	2004–2005*	0.94	0.13	0.20 to 1.0
	2005–2006	0.85	0.17	0.30 to 0.99
	2006–2007	0.94	0.15	0.08 to 1.0
	2007–2008*	0.68	0.24	0.20 to 0.95
Capture probability	2004*	0.51	0.38	0.05 to 0.96
	2005	0.22	0.05	0.14to 0.33
	2006	0.19	0.05	0.11 to 0.32
	2007	0.22	0.05	0.14 to 0.33
	2008*	0.18	0.07	0.08 to 0.34
Superpopulation		404.9	44.9	316.8 to 1492.9
Net Abundance	2004*	160.0	88.6	-13.7 to 333.6
	2005	204.5	46.2	114.0 to 295.0
	2006	264.4	81.2	105.3 to 423.5
	2007	347.3	70.7	208.7 to 485.9
	2008*	239.3	110.1	23.52 to 455.07
Entry probability	2005–2006	0.23	0.17	0.04 to 0.67
	2006–2007	0.25	0.15	0.06 to 0.62

* The full model φ(t),*p*(t),*b*(t) was excluded from model averaging for the first and last estimates due to parameter confounding [48].

### Point surveys

Hellbenders were encountered at 19 of 23 sites over 28 river km, and capture rates varied from 0 to 4 individuals per person hour (mean = 1.28, 95% CI = 0.80–1.76). The 2009 point survey capture rate at the CMR site was 4.6 hellbenders per person hour. Using the actual density estimate of 84 hellbenders per 100 m of river length calculated above from the 2004–8 CMR site, the average density between the Apalachia powerhouse and Patty Bridge was (84/4.6)*1.28 = 23 (95% CI = 15–31) hellbenders of taggable size per 100 m, indicating a total population of 6440 (95% CI = 4200–9240) taggable sized hellbenders over 28 river km.

### Public lands

**Fig 4:**
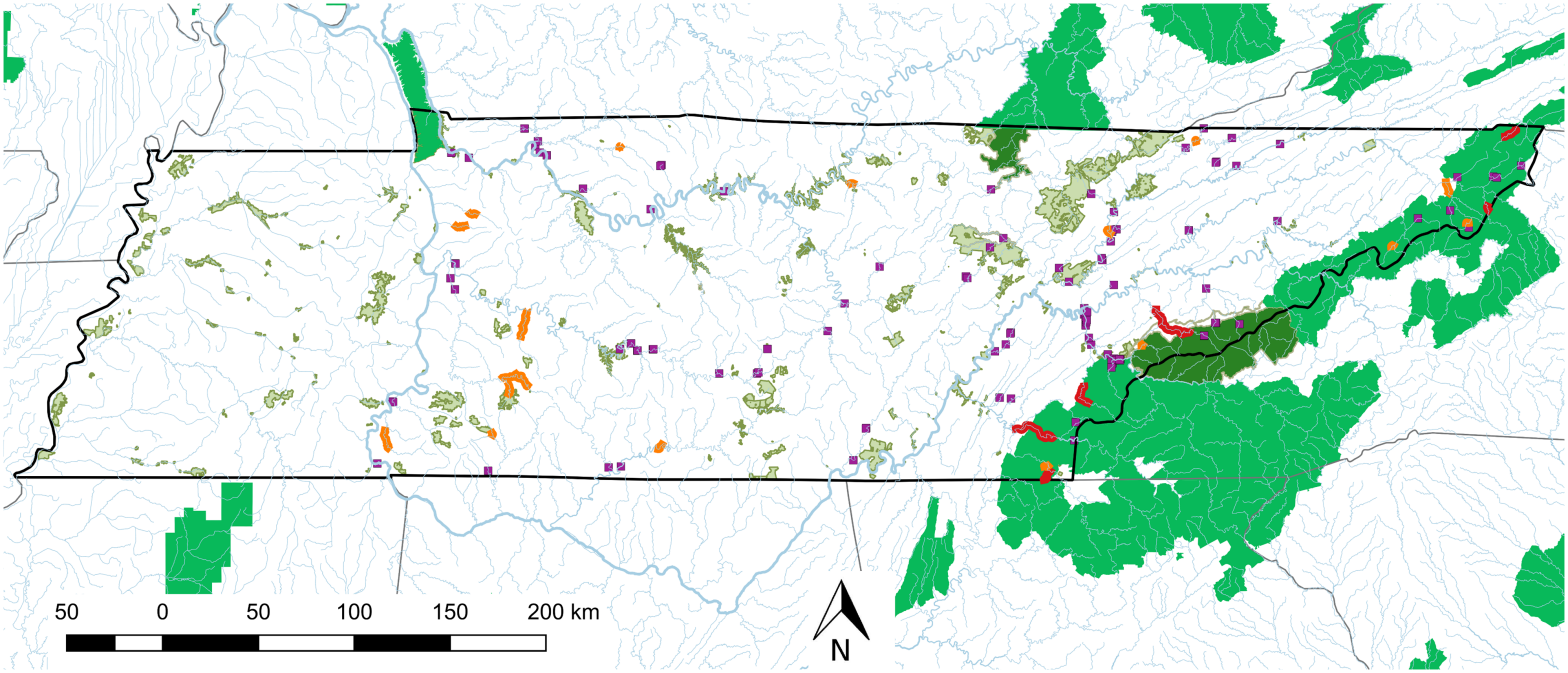
Map of public lands overlaying contemporary and historic eastern hellbender populations in Tennessee. Shaded areas show National Park (dark green), National Forest (mid green), and State Wildlife Management Areas (light green). Purple squares indicate locations for which there are historic (1915–2000) eastern hellbender occurrences, but no contemporary (post 2000) sightings. Orange and red lines indicate the extent of contemporary eastern hellbender populations based on verified captures from 2001 to 2015. In the red populations, a range of age classes including larvae, juveniles and adults have been observed during this period, while in orange populations only adults have been documented.

We identified a minimum of 54 separate rivers and streams with hellbender occurrence records since 1915 (Fig 4). However, because we pooled data from multiple sites on the same river that in some cases was very long (e.g. 272 km for the Cumberland River), this is likely an underestimation of the number of historic populations. Only 21 of these rivers and streams have verified observations of hellbenders since 2000, representing a decline of 61%. These occurrences comprise about 200 river km in total. Of the 21 rivers, 10 are entirely disconnected from federal public lands, while 11 lie partially or entirely within federal public lands, and one middle Tennessee population lies partially within state public lands. There are just 6 populations with larvae, juveniles and adults present, which total 174 river km, and of that ~60 km lie within or border federal public lands. All recent (since 2000) observations of larval hellbenders in Tennessee occur within those ~60 km.

## Discussion

Our study indicates the Hiwassee River has a substantial population of hellbenders with consistent successful reproduction each year. On average, gilled larvae represented 21.5% of all captures which is likely to be an underestimate given their small size and lower detectability compared to subadults and adults. Reproduction in the Hiwassee occurs in October, and larval hellbenders remain under the nest rock guarded by a male until apparently dispersing in late April (Jeff Humphries, North Carolina Wildlife Resource Commission, pers. comm.; M. J. Freake, Lee University, pers. obs.). The next size class (9–12 cm) only made up 2% of captures. Assuming there was no net emigration of larval hellbenders within the study site over the first year, mortality rates of larvae post dispersal are ~ 90%, which is consistent with estimates for combined egg/larval survival provided by Unger et al [16]. Also, they used Lefkovitch stage structured matrices to show that a stable stage distribution should be characterized by a very high (~84%) egg/larva stage frequency. In other words, high levels of reproduction and recruitment are essential to maintain population stability, and so the high frequency of hellbender larvae observed in the Hiwassee indicates a stable, viable population.

Size class frequencies remained similar until growth rates slowed until around 30 cm where cohorts become indistinguishable and size class frequencies increase (see Fig 2) suggesting that if a hellbender can survive its first year of life, survival probabilities are high through the subadult and adult phases, which is supported by our high apparent survival rate estimates (68–94%). It is interesting that the lowest survival rate estimate corresponded to the last year of the study (2007–2008). It is possible that repeated rock lifting may increase stress [3], and so it is conceivable that the four years of prior surveys may have increased local mortality and/or emigration rates, which would cause a decrease in apparent survival.

There are several factors that contributed to our success in detecting larval hellbenders. First, there appears to be consistent successful reproduction each year in this population. Second, the Hiwassee water clarity is excellent, and the shallow cobblestone substrates are relatively amenable to systematic searches. Third, our surveys were conducted primarily in May within weeks of emergence from the nest rock, giving little time for dispersion and predation to reduce abundance. Conversely, most previous studies did not detect larval hellbenders, presumably because of significant differences in recruitment rates, substrate type, or survey technique. It is possible that declines had already commenced by the mid 1970’s and the lack of larval captures was symptomatic of poor recruitment. However, substrate type is a more likely explanation: Nickerson et al [49] argued that historic Ozark sites had large expanses of deep gravel beds with open interstices making larval hellbenders almost impossible to find, while the high frequency of larval hellbenders encountered in Little River in the Great Smoky Mountain National Park (GSMNP) was due to a lack of gravel deposits forcing larvae to refuge under cobblestones and boulders where they could be easily encountered during snorkel rock turning surveys. Like Little River, the Hiwassee is dominated by metamorphic sandstone and siltstone rock which tend to produce a thin layer of small rounded pebbles and cobblestones rather than the thick layers of chert, dolomite and limestone gravel characteristic of Ozark rivers. Also, differences in survey technique can dramatically alter the probability of catching larval hellbenders [39], which was strongly supported by our surveys in the Hiwassee; we ignored small rocks when conducting point surveys, and larval hellbenders composed just 3% of captures.

We were interested in comparing the density of hellbenders in the Hiwassee with other pre- and post-decline populations (see Table 4). We determined that peak density was 2.31 taggable sized hellbenders per 100 m^2^ of suitable habitat or 840 hellbenders per river km, with an average density of 230 hellbenders per river km. Our population estimates are derived from five consecutive years of survey data. Five years is significantly longer than all other published hellbender CMR studies, which were conducted over either one or two years. Therefore, our estimates should be less sensitive to errors caused by stochastic events, which might temporarily influence local abundance, or affect search efficiency and encounter probabilities [50]. Previous studies produce highly divergent estimates of densities, and differences in methodology can make comparisons difficult. For example Bothner and Gottlieb [20] and Foster et al. [22] used closed population Lincoln-Peterson methods in Allegany river tributaries to estimate densities of 10–60 and 0–20 hellbenders per 100 m^2^ of suitable habitat respectively. However, they defined habitable spaces as estimated square meters of cover rock in the study area, and thus only 2–10% and 1–6% respectively of the streambed was considered suitable habitat. Nickerson and Mays [17] reported an estimate of 428 hellbenders per km over a 2.67 km length of the North Fork of the White River (Ozark), with a peak density of 269 hellbenders in a 4600 m^2^ riffle and estimated 50–60% as “suitable diurnal habitat” giving “one hellbender per 8–10 m^2^” (10–13 hellbenders per 100 m^2^). Similar densities were observed during surveys at the same site a decade later [13]. Estimates from the Spring, Eleven Point Gasconade and Big Piney in 1982 varied from 1 to 6 hellbenders per 100 m^2^ total area [12], and the authors believed these densities were similar to those of Nickerson and Mays [17] because they included the entire section of river rather than just ideal habitat. The peak density we observed in the Hiwassee is about a quarter of the peak density observed by Nickerson and Mays [17], illustrating the remarkable historic density of hellbenders at their study site in the North Fork of the White River. We determined the Hiwassee CMR site has particularly high density compared to other sections of the river, and the estimate of 15–33 hellbenders per 100m averaged over the 28 km of occupied river is about half of the typical densities observed in the Ozarks prior to declines. None of the original studies report capture rates, although Nickerson et al [33] state the capture rates were 8–12 per person hour. It is not surprising that Ozark rivers tended to have higher hellbender densities than the Hiwassee considering the substantial differences in alkalinity and associated productivity [49], with food rather than suitable shelter rocks likely being a limiting factor.

**Table 4:** Comparison of studies estimating eastern hellbender population densities or relative abundances, organized by date of publication. Densities are expressed as number of hellbenders per 100 m^2^ of river. Relative abundance is given by catch per unit effort (CPU), which is the number of hellbenders caught per person hour searching. Adapted with permission from Burgmeier et al. [21].

Date	Authors	Density	CPU	State
1971	Hillis and Bellis [23]	0.99/100 m^2^ ^a^		PA
1973	Nickerson and Mays [17]	10-13/100 m^2^	8–12	MO
1984	Kern [51]	20.2 ±7.7/100 m ^b^		IN
1988	Peterson et al. [12]	1-6/100 m^2^		MO
1991	Bothner and Gottlieb [20]	0.32–3.73/100 m^2^, (5.75–58.82/100 m^2^) ^c^	0.05	NY
2002	Nickerson et al. [33]		0.25–0.65	TN
2003	Wheeler et al. [8]		3.3–10 ^d^	MO
2005	Humphries and Pauley [52]	0.8–1.2/100 m^2^		WV
2009	Foster et al. [22]	0–1.07/100 m^2^, (0-20/100 m^2^) ^c^		NY
2011	Burgmeier et al. [21]	0.06/100 m^2^		IN
2012	Hecht-Kardasz et al. [32]		0.34	TN
2013	Pugh et al. [34]		0.83	TN
2017	Freake and Deperno	2.3/100 m^2^	0–4.6	TN

^a^ Estimated from original data of individuals captured within study area.

^b^ This estimate is per 100 meters of river length. Kern did not provide sufficient stream width data to allow estimation of density per unit area.

^c^ Values in parentheses are the reported densities per 100 m^2^ of “suitable habitat” only.

^d^ No river distance, area, or search effort values were reported. These are the mean number of hellbenders captured per day.

Our capture rates and density estimates of eastern hellbenders in the Hiwassee appear to be much higher than those observed in recent post-decline studies of other populations across their range. Burgmeier et al. [21] detected an average density of 0.06 hellbenders per 100 m^2^ (total area) in the Blue River in Indiana, with a CPU of 0.05 hellbenders per hour which represents a significant decline compared to Kern [51] who estimated densities of 20 hellbenders per 100 m of streambed. Other recent studies within the Blueridge province report observed densities of 0.8 per 100 m^2^ in a tributary of the New River in West Virginia [52], and capture rates varying from 0.25 to 0.83 hellbenders per person hour in Little river in the Great Smoky Mountains National Park [32,33] and the Watauga [34] respectively.

Our study provides an important reference point for long term monitoring of eastern hellbenders in Tennessee. Without baseline data from 1970 to 1990, it would have been difficult to document the decline of hellbenders in the Ozarks [8,12,17]. Moreover, the population size and high rates of recruitment suggest the Hiwassee represents one of the most significant contemporary populations of eastern hellbenders in the range. In addition, Hiwassee hellbenders exhibit the highest diversity of mitochondrial haplotypes and microsatellite alleles of any of the populations in the Tennessee River system (Freake et al., unpublished data). Possible threats to the population include changes in hydroelectric generation schedule and shifts in public perception of the use and value of public lands.

We believe that National Forest and National Park lands are extremely important to the ongoing management and recovery of hellbenders in Tennessee. Historic occurrence records support the statement by Gentry [30] that eastern hellbenders were widespread and abundant in Tennessee in the mid 20th century. However, since 2000, there are just 21 populations with verified observations of hellbenders in Tennessee, comprising about 200 river kilometers. Many of the declines are likely attributable to large sections of historic habitat being inundated by impoundments (e.g. Cumberland and Little Tennessee rivers), or to significant water quality declines from land use changes over much of their length (e.g. Duck river). In contrast, watersheds that lie within National Forest and National Park public lands have very high percent forest cover and low rates of anthropogenic sources of sediment. As a result, water quality is high and large unembedded rock substrate [26,49,53] is abundant. As expected, we found a strong association between public lands and eastern hellbender populations that currently show evidence of consistent reproduction and recruitment. We estimate there are now just 60 river kilometers in Tennessee that appear to show consistent reproduction and recruitment, and all of those 60km lie within or border federal public lands. The Hiwassee is the most significant of these, with 17 river kilometers characterized by high hellbender densities, lying within the Cherokee National Forest. Hellbenders are not the only species to benefit from the high water quality found in the Cherokee National Forest and Great Smoky Mountains National Park; 31 species of salamander have been recorded in the Great Smoky Mountains National Park [54], and including hellbender there are three salamander species and five fish species [55] that are federally listed or a species of concern found within these public lands.

One noticeable feature is the absence of hellbenders from numerous Tennessee Valley rivers on public lands that appear to have excellent water quality and suitable habitat. We believe these rivers are impacted by historic and contemporary land use practices at multiple hierarchical levels within the watershed. Impoundment and impairment of lower-mid elevation mainstem river sections has caused extensive fragmentation and isolation of populations on public lands, which tend to be close to the upper elevation limit for hellbenders. Prior to the establishment of the Cherokee NF and GSMNP, these populations experienced severely destructive practices such as mining and large scale clear-cutting including splash dam logging [56], causing many historic populations to be extirpated at the time, or at best to become small and isolated. Demographic and environmental stochasticity and limited opportunities for natural rescue through migration could then have increased the risk of extirpation [57], even while the watersheds were being managed for recovery as National Forest and National Park public lands. A management plan for hellbenders in Tennessee should include translocations of individuals from healthy populations or a captive breeding head-start program, to rivers in protected watersheds with declining or extirpated hellbender populations. Habitat modeling would be valuable not only in identifying appropriate target rivers, but also in identifying parameters associated with healthy populations which could then be used to guide restoration programs for impaired rivers.

## Supporting information

S1 Appendix Study area. (DOCX)

S1 Fig Study site. Photograph illustrates a portion of the CMR study site comprising of shallow cobblestone glides (foreground) interspersed with deeper runs and riffles containing large shelter rocks.
(DOCX)
