# Supplementary material for: Importance of demographic surveys and public lands for the conservation of eastern hellbenders Cryptobranchus alleganiensis alleganiensis in southeast USA
Source: PLoS One. 2017 Jun 8;12(6):e0179153. doi: 10.1371/journal.pone.0179153 (PMC5464636; doi:10.1371/journal.pone.0179153)
Supplement: S1 Appendix — (DOCX) [file pone.0179153.s001.docx]

**S1 - Study Area**

The headwaters of the Hiwassee River begin in the Chattahoochee National Forest in North Georgia, and the mainstem flows into North Carolina and Tennessee before joining the Tennessee River at Chickamauga reservoir. The river is impounded by four Tennessee Valley reservoirs within North Carolina (Chatuge, Mission, Hiwassee and Apalachia dams). The Hiwassee watershed drains over 6400 km of streams and encompasses about 7000 km^2^. Approximately 30% is above 500m elevation with almost 90% of the watershed consisting of mountainous, forested topography, of which one third belongs to the US Forest Service.

Flows below Apalachia powerhouse typically vary from 6 to 80 m^3^/sec, but additional water may be spilled from the dam during high rain periods. The river between the powerhouse and Hwy 411 is characterized by extensive pools and shoals created by exposed inverted bedrock strata, linked by runs and riffles. Erosion of these strata produces numerous pieces of bedrock that provide excellent shelter rocks for hellbenders. Habitat is primarily limited by water depth and flow – some shallow areas are scoured by the water releases preventing accumulation of unembedded shelter rocks, while other areas are deep and slow with shelter rocks tending to become embedded by sediment. The rock substrate is composed of metamorphosed sedimentary siltstones, sandstones, and shale from the Blue Ridge physiographic province [36] and the lack of limestone results in soft water conditions and low alkalinity (< 12 mg/L CaCO_3_, pH ~ 6.9) ([37]; M. J. Freake, Lee University, unpublished data: hardness was measured at using a Hach model 5EP MG-L total hardness test kit, pH was measured using a Hach model 17F pH test kit). Below Hwy 411 the river enters the ridge and valley physiographic province, characterized by higher levels of limestone and lower gradient (Hwy 411 to Patty Bridge gradient = 0.9 m/km, Hwy 411 to powerhouse = 2.6 m/km). The bedrock ledges tend to disappear and the substrate is dominated by gravel and cobblestone with isolated pieces of bedrock and fewer shelter rocks.
