# Supplementary material for: Importance of demographic surveys and public lands for the conservation of eastern hellbenders Cryptobranchus alleganiensis alleganiensis in southeast USA
Source: PLoS One. 2017 Jun 8;12(6):e0179153. doi: 10.1371/journal.pone.0179153 (PMC5464636; doi:10.1371/journal.pone.0179153)
Supplement: S1 Fig — Photograph illustrates a portion of the CMR study site comprising of shallow cobblestone glides (foreground) interspersed with deeper runs and riffles containing large shelter rocks. (DOCX) [file pone.0179153.s002.docx]

**S1 Fig. Study Site.**

**
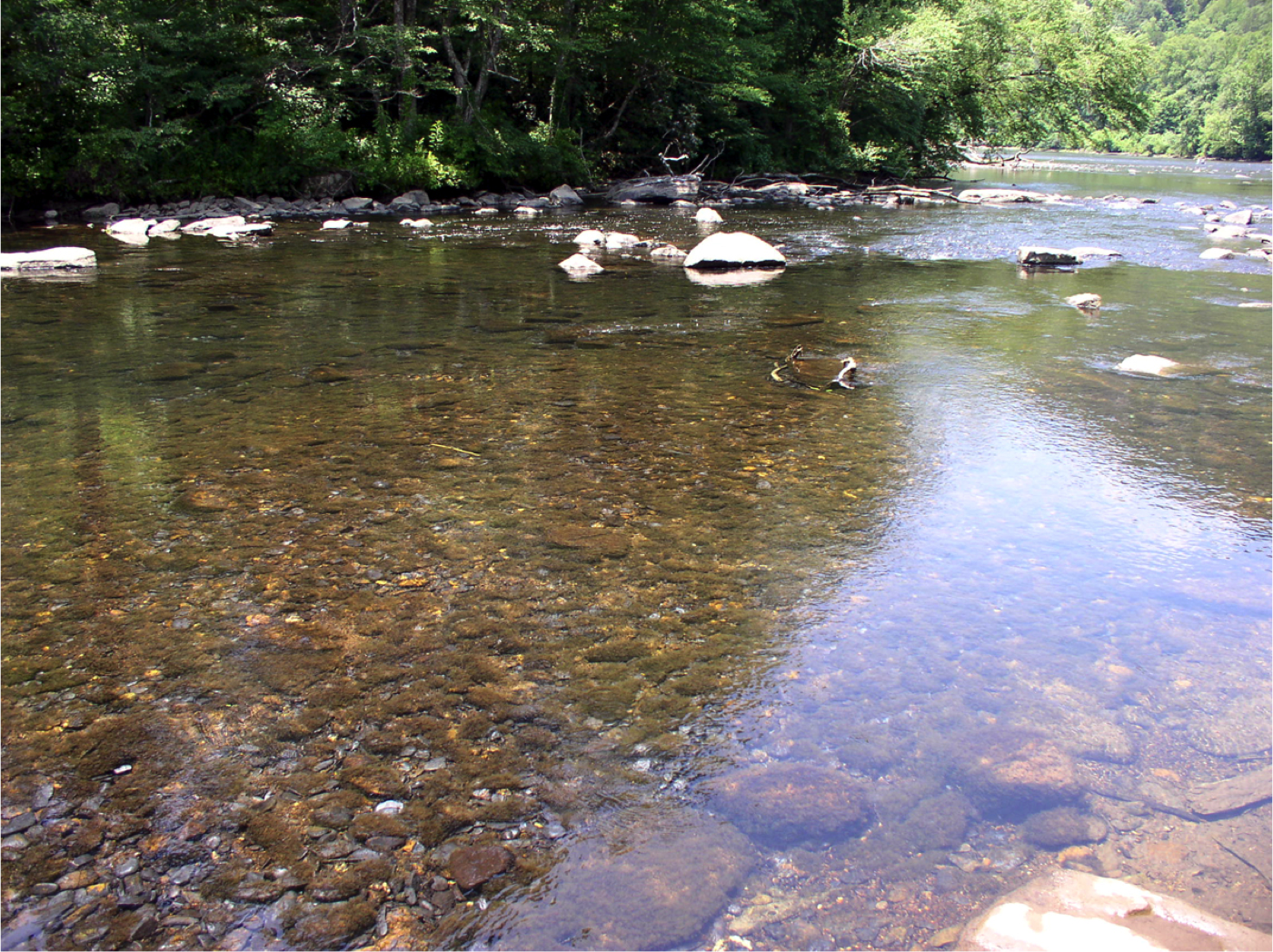
**Photograph illustrates a portion of the CMR study site comprising of shallow cobblestone glides (foreground), and deeper runs and riffles containing large shelter rocks (background)
